# Posterior Protrusion Measures (PPM) for Three-Dimensional (3D) CT Classification of Pertrochanteric Fractures

**DOI:** 10.7759/cureus.51363

**Published:** 2023-12-30

**Authors:** Mitsuaki Noda, Shunsuke Takahara, Atsuyuki Inui, Keisuke Oe, Shin Osawa, Takehiko Matsushita

**Affiliations:** 1 Orthopedics, Nishi Hospital, Kobe, JPN; 2 Department of Orthopedics, Hyogo Prefectural Kakogawa Medical Center, Kakogawa, JPN; 3 Department of Orthopedic Surgery, Kobe University Graduate School of Medicine, Kobe, JPN; 4 Department of Orthopedics, Himeji Saint Mary's Hospital, Himeji, JPN

**Keywords:** subclassification, unstable, stable, greater trochanter, posterior protrusion measures, pertrochanteric fractures, 3d ct classification

## Abstract

Introduction

We introduced a novel numerical index known as posterior protrusion measures (PPM), derived from lateral plain radiograph images, which effectively serves to distinguish stable from unstable pertrochanteric fractures. The present study aims to scrutinize PPM values among two classified fracture patterns, stable and unstable, within the three-dimensional (3D) CT classification system, establishing a numeric threshold for PPM to differentiate between these groups; explore the potential relationship between the PPM index and unclassified categories; investigate how groups divided by the PPM threshold value can predict fracture stability based on 3D CT.

Materials and methods

In this study, three observers were tasked with measuring PPM on a single occasion. The chi-square test assessed the association between each demographic parameter on a categorical scale and stable/unstable groups. Continuous variables were also subject to examination. Receiver operating characteristic (ROC) analysis was employed to determine optimal cut-off points of PPM for predicting the presence of stable versus unstable groups. Additionally, the chi-square test examined the linear relation between separated groups based on the defined threshold PPM value and the stable/unstable groups.

Results

A total of 106 pertrochanteric fractures were identified using CT scan images and plain radiographs in the 3D CT classification system, revealing the stable group of 35 patients and the unstable group of 71 patients. The PPM values for stable/unstable fractures were, on average (± standard deviation), 0.34±0.25/0.50±0.29 for observer 1, 0.31±0.23/0.57±0.31 for observer 2, and 0.41±0.29/0.57±0.26 for observer 3, respectively (p<0.01). We established 0.3 as the cut-off value for PPM. The average PPM value among three observers represented each patient to assess fracture stability. The group with PPM <0.3 included 27 patients (16 stable and 11 unstable), and the group with PPM ≥0.3 group comprised 79 patients (19 stable and 60 unstable; p<0.005).

Conclusion

The present study revealed a significant difference in PPM values among stable and unstable 3D CT classification groups. Additionally, a threshold PPM value of 0.3 suggests a pivotal point for differentiating fracture stability. This innovative methodology makes a substantial contribution to clinical endeavors, potentially circumventing the necessity for 3D CT scanning.

## Introduction

The incidence of femoral pertrochanteric fractures in the elderly population has risen, especially in developed countries [1]. Obtaining a precise characterization of these fractures holds the potential to alleviate burdens across various clinical scenarios. Firstly, accurate differentiation between stable and unstable fractures significantly influences the choice of implant for procedures such as dynamic hip screwing (DHS), intra-medullary nails (IMN), or a modified DHS [2-4]. Noteworthy reports indicate that opting for IMN over DHS in unstable cases may result in improved functional outcomes [5,6]. Secondly, a meticulous depiction of the trauma facilitates the intra-operative reduction of displaced fragments in the greater trochanter [7]. Thirdly, epidemiological studies classifying pertrochanteric fractures bimodally gain heightened reliability [8,9]. Therefore, accessing accurate three-dimensional (3D) images of pertrochanteric fractures becomes a valuable pursuit.

With the advancement of modern technology, computed tomography (CT) scan images adeptly capture the intricate morphology of fractures in the posterior intertrochanteric region [10-12]. However, routine use of CT scans in elderly patients with femoral trauma proves challenging due to limited accessibility and cost, particularly in developing countries. Additionally, the associated drawback of excessive radiation exposure poses a significant concern [13]. Thus, if interpretations based on plain radiographs can provide a glimpse into 3D CT images, this method could offer benefits to surgeons and patients globally.

In our preliminary study, we introduced an innovative numerical index referred to as posterior protrusion measures (PPM) [14]. Utilizing lateral plain radiograph images among different rotational positions, this method effectively distinguishes A1 (stable) from A2 (unstable) fractures according to the revised Arbeitsgemeinschaft für Osteosynthesefragen (AO Foundation)/Orthopedic Trauma Association (AO/OTA) Classification in 2018 [15]. This approach proves to be both simple and valuable, providing orthopedic surgeons with a tool for classifying pertrochanteric fractures. We anticipate that it may also find application in 3D graphic analysis. The present ambitious study aims to (i) scrutinize PPM values among two classified fracture patterns, stable or unstable, under the 3D CT classification system and establish a numeric threshold for PPM to differentiate these groups; (ii) investigate the potential relationship between the PPM index and subclassified categories, to assist surgeons in recalling 3D pictures of displacement; (iii) explore how divided groups with the threshold value of PPM can predict fracture stability based on 3D CT. We hypothesize that employing the PPM method will reveal significant differences in PPM values between 3D CT-based stable and unstable groups, potentially contributing substantively to the delineation of graphical fracture type within the 3D CT sub-classification system without a CT scan.

## Materials and methods

Data source and collection

Patients diagnosed with pertrochanteric fractures between January 2019 and December 2020 were retrospectively identified using the keyword "pertrochanteric fracture" in the surgical database of two hospitals: Kasai Municipal Hospital and Suzuran Hospital. Enrollment criteria necessitated both anteroposterior and lateral plain radiographs, along with CT scans (3D CT). Exclusion criteria encompassed assumed subtrochanteric fractures, pathologic fractures, the presence of moderate or severe osteoarthritis, and a history of previous ipsilateral hip fracture.

**Figure 1:**
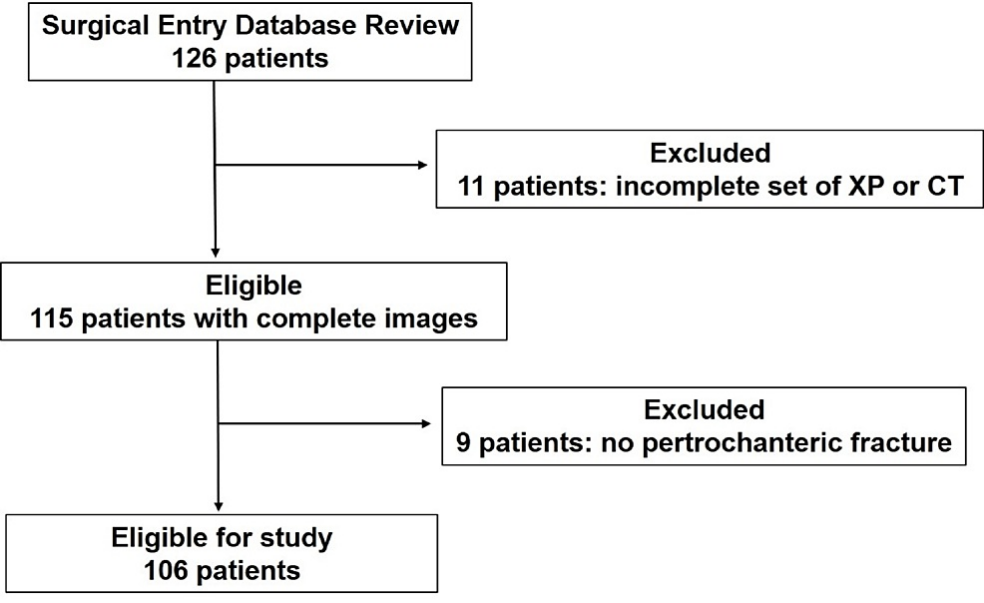
Flow diagram for patient selection XP - plain radiograph

Out of the initially identified 126 patients, 20 were excluded due to incomplete graphic images (11 patients) and the absence of pertrochanteric fractures (nine patients). Consequently, 106 patients (17 male, 89 female) were included in the study (Figure 1). The mean age at the time of surgery was 87 years (ranging from 50 to 99 years), with fractures occurring on the left side in 60 cases and the right side in 46.

Fracture classification

The 3D CT classification by Shoda et al. [11] comprises seven categories: a two-part, three-part G(S), three-part G(B), three-part G-L, three-part G(W), three-part L, and four-part, in combination with four major fragments of the femoral head, greater trochanter (G), lesser trochanter (L), and shaft. A two-part is simple where G and L are intact. A three-part G(S), with the small greater trochanteric fragment, where S indicates small. A three-part G(B) indicates a big oblique fragment of the greater trochanter that does not include the lesser trochanter, where B means big. A three-part G-L involves a large oblique fragment of the greater trochanter with the lesser trochanter. A three-part G(W) accompanies a fragment of the whole greater trochanter as W is shortened for whole, but the lesser trochanter is intact. A three-part L is with a non-fractured greater trochanter. A four-part revealing both the greater trochanter and lesser trochanter are fractured and displaced, respectively.

In the present study of 3D CT classification of pertrochanteric fracture, observer 1 classified pertrochanteric fractures into two groups: stable group for two-part and three-part G(S), and unstable group for the remaining, based on personal communication with Shoda. Whereas, their original article defined stable groups for two-part, three-part G(S), and three-part G(B), and unstable groups for others. Additionally, sub-classification was composed of seven categories depending on major fragments.

Posterior protrusion measures (PPM) using plain lateral radiograph images

**Figure 2:**
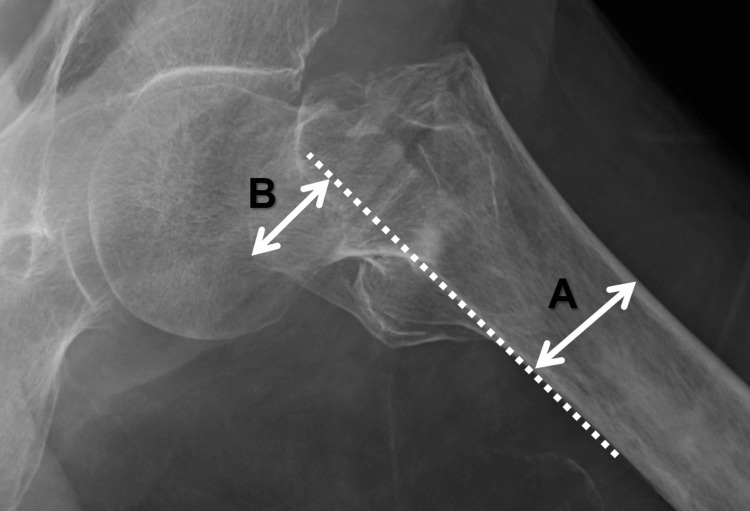
Posterior protrusion measure (PPM): a value of posterior protrusion of the greater trochanter PPM serves as an eloquent indicator of the posterior displacement of the greater trochanter, providing a quantitative measure of the severity of pertrochanteric fractures. The formula governing PPM is elegantly expressed as PPM=B/A, where A denotes the femoral diameter, B signifies the length of the displaced greater trochanter, and the broken lines indicate a trajectory along the posterior edge of the proximal femur.

Two senior orthopedic surgeons (observer 1 and observer 2) and one resident (observer 3) conducted PPM measurements, determining the value of posterior displacement of the greater trochanter divided by the diameter of the proximal femoral shaft (Figure 2). Each observer worked independently, adjusting image size, brightness, and contrast for accurate osseous structure identification. A one-month lag was maintained between the 3D CT classification using CT images by observer 1 and the PPM evaluation. The PPM technique was thoroughly detailed in a previous article [14].

Statistical analysis

Data analysis utilized EZR (Saitama Medical Center, Jichi Medical University, Saitama, Japan), a graphic user interface for R (The R Foundation for Statistical Computing, Vienna, Austria), with a significance threshold set at p<0.05. The chi-square test assessed demographic parameters between stable and unstable groups. Continuous variables were presented as mean ± standard deviation, while categorical variables were expressed as numbers (n) and percentages (%). The Kolmogorov-Smirnov test confirmed data normality.

Interclass correlation coefficient (ICC) values were calculated for inter-observer agreement, interpreted as poor (less than 0.00), slight (0.00-0.20), fair (0.21-0.40), moderate (0.41-0.60), substantial (0.61-0.80), and almost perfect (0.81-1.00) [16].

For PPM value evaluation between stable and unstable groups, the independent sample t-test or Mann-Whitney U test was applied based on data normality. Receiver-operating characteristic (ROC) analysis determined optimal cut-off points of PPM, with the area under curve (AUC) categorized as excellent (0.9-1), good (0.8-0.89), fair (0.7-0.79), poor (0.6-0.69), or fail/no discriminatory capacity (0.5-0.59) [17]. Jonckheere-Terpstra's test examined incremental changes in the seven consecutive sub-classified groups.

Additionally, the chi-square test was used to examine the association between the stable and unstable groups and groups classified with the designated cut-off point of PPM. Confidence intervals (CI) were calculated for the three observers using a sample size of 60 patients (lower limit: 0.5, upper limit: 0.8, α=0.05) [10]. Employing the post hoc power analysis, we gauzed the appropriateness of our sample size.

Ethics, funding, and potential conflicts of interest

This study received approval from the research ethics board of each participating hospital (represented by the Nishi Hospital IRB committee with approval number 2021-1). No competing interests were declared, and the study did not receive external funding.

## Results

Comparison of demographic variables between stable and unstable fractures

A comprehensive examination encompassed 106 pertrochanteric fractures identified through meticulous analysis of CT scan images and plain radiographs. Initial categorization led to the formation of two distinct groups within the 3D CT classification system: stable and unstable.

The stable group, comprised of 35 patients (6 males [M], 29 females [F]), was juxtaposed against the unstable group, consisting of 71 patients (11 M, 60 F) with no significant (NS) gender distribution disparities. Age at the time of surgery exhibited marginal differences, with the stable group averaging 87.9±5.7 years (range: 76 to 98 years old) and the unstable group at 87.4±8.0 years (range: 50 to 99 years old; NS). However, the laterality of the fracture site revealed a significant distinction, with 14 left and 21 right cases in the stable group and 46 left and 25 right cases in the unstable group (p<0.05; see Table 1).

**Table 1:** Comparison of demographic variables between stable and unstable fractures of 3D CT interpretation †^ ^mean age and standard deviation; ‡ p-value for two groups analyzed by gender and laterality of fracture site; ※ p-value for age groups

Variables	Stable n=35	Unstable n=71	p-value
Male, n(%)	6 (17.1%)	11 (15.5%)	0.84‡
Female, n(%)	29 (82.9%)	60 (84.5%)	
Age at surgery† (years)	87.9±5.7 (range: 76-98)	87.4±8.0 (range: 50-99)	0.71※
Laterality: left	14 (40%)	46 (64.8%)	0.015‡
Laterality: right	21 (60%)	25 (35.2%)	

Subclassification into seven categories included two-part (15 patients, including 3 M, 12 F), three-part G(S) (20 patients, including 3 M, 17 F), three-part G(B) (12 patients, including 12 F), three-part G-L (54 patients, including 9 M, 45 F), three-part G(W) (three F patients), three-part L (one F patient), and four-part (one M patient).

PPM values inter-observer agreement

In assessing inter-observer agreement, the PPM intra-class correlation coefficient (ICC) values were determined. These values reflected substantial to perfect inter-observer agreement: 0.81 (0.733-0.867) between observers 1 and 2, 0.624 (0.493-0.727) between observers 2 and 3, and 0.764 (0.654-0.839) between observers 1 and 3.

Evaluation of PPM values between stable and unstable groups and among subclassification

Analysis of PPM values across observers showcased significant differences between the stable and unstable groups. Specifically, the stable group consistently exhibited lower PPM values compared to the unstable group (p<0.01; see Table 2).

**Table 2:** PPM values in the stable and unstable groups of 3D CT interpretation ‡^  ^independent sample T-test Observers 1 and 2 were experienced orthopedic surgeons, and observer 3 was an orthopedic resident. PPM - posterior protrusion measures

Observers	Stable n=35	Unstable n=71	p-values
1	0.34±0.25	0.50±0.29	0.006‡
2	0.31±0.23	0.57±0.31	<0.001‡
3	0.41±0.29	0.57±0.26	<0.01‡

Further, within the sub-classification, PPM values displayed incremental variations. The average values ± standard deviation of PPM for the different categories were observed, as shown in Table 3.

**Table 3:** The average values ± standard deviation of PPM for the different categories according to observers

Observers	Two-part	Three-part G(S)	Three-part G(B)	Three-part G-L	Three-part G(W)	Three-part L	Four-part
1	0.27±0.27	0.39±0.24	0.42±0.33	0.49±0.24	0.84±0.68	0.88	0.88
2	0.29±0.24	0.33±0.23	0.48±0.28	0.55±0.27	0.89±0.69	0.69	1.35
3	0.36±0.35	0.44±0.24	0.50±0.29	0.55±0.21	0.67±0.65	0.86	1.1

**Figure 3:**
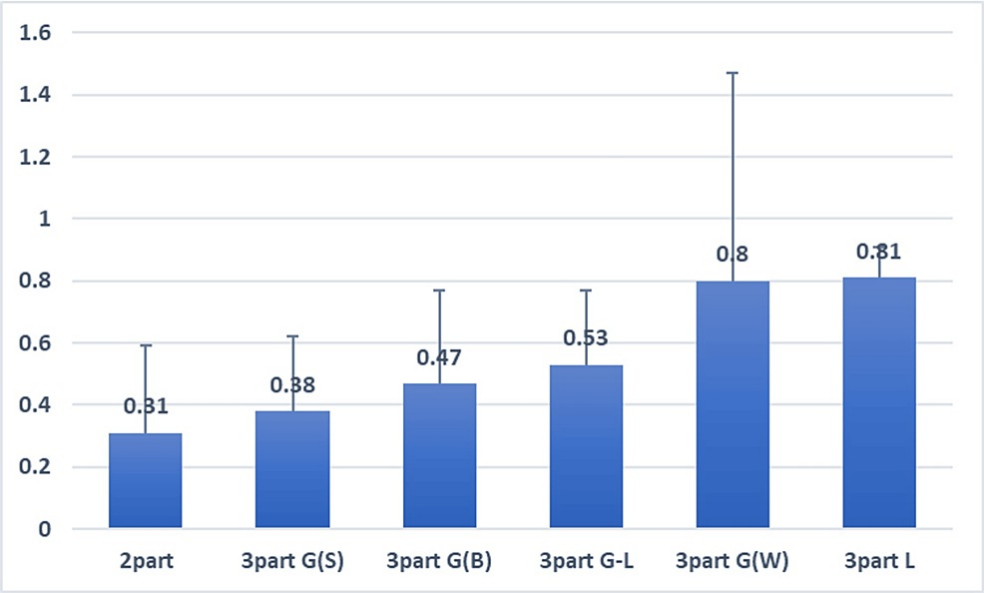
Stepwise increase in posterior protrusion measures (PPM) among seven subclassified groups The graphical representation delineates a stepwise escalation in average PPM values, accompanied by standard deviations, across all observations. As the 3D CT classification progresses through the stages of two-part, three-part G(S), three-part G(B), three-part G-L, three-part G(W), three-part L, and four-part, a statistically significant incremental variation in PPM values is discerned (p<0.01, Jonckheere-Terpstra test).

The median value of PPM was 0.53 (range: -0.12-1.71). Notably, a discernible trend toward higher PPM with higher Shoda 3D CT categories was observed in this examination (p<0.01, Jonckheere-Terpstra test; Figure 3).

Receiver operating characteristic (ROC) curve to determine the cut-off point

**Figure 4:**
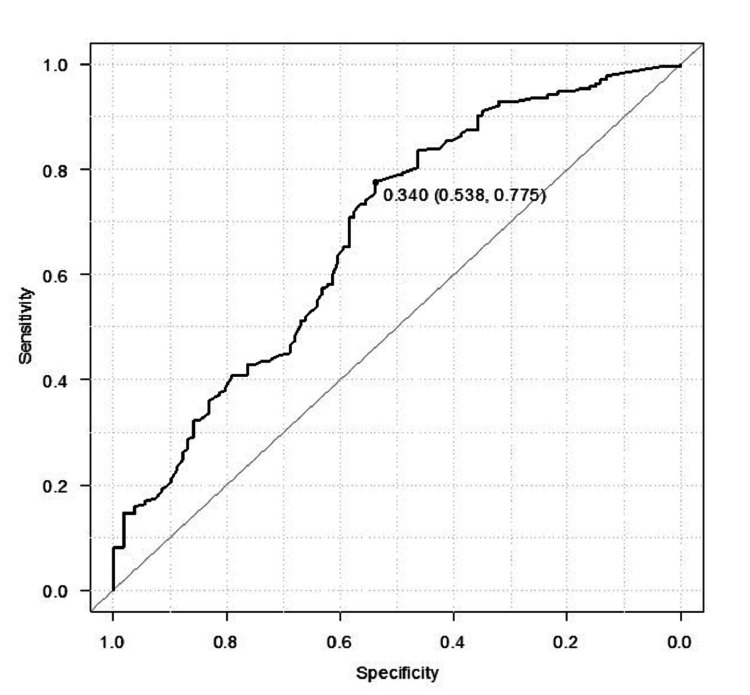
Receiver operating characteristic curve showing the sensitivity against the 1–specificity The receiver operating characteristic (ROC) aptly encapsulates the delicate balance between sensitivity and specificity. The area under the curve (AUC: 0.678, 95% CI: 0.614–0.741) serves as a quantitative testament to the PPM value's proficiency in accurately categorizing patients into stable and unstable groups within the framework of 3D CT fracture classification. The optimal cut-off point, determined through Youden's index, is 0.34. PPM - posterior protrusion measures

The receiver operating characteristic (ROC) curve was employed to ascertain the cut-off point for PPM value in distinguishing stable versus unstable fracture groups. The identified cut-off PPM values were 0.333 for observer 1, 0.300 for observer 2, and 0.390 for observer 3, exhibiting acceptable sensitivity and specificity (Table 4). At the averaged cut-point level of 0.34 among the three observers, the unstable group demonstrated sensitivity, specificity, positive predictive values, and negative predictive values of 77.5%, 53.8%, 45.7%, and 22.9%, respectively (AUC: 0.678, 95% CI: 0.614-0.741; see Figure 4). Thus, we simplified the cut-off value to 0.3, dichotomizing pertrochanteric fractures into stable and unstable groups under the 3D CT classification system.

**Table 4:** Cut-off PPM values to access unstable groups classification The calculated cut-off values, guided by Youden’s index, intricately delineate the classification of stable and unstable groups based on PPM values. PPM - posterior protrusion measures; AUC - area under curve; CI - confidence interval

Observers	AUC (CI: range)	Cut-off	Sensitivity	Specificity
1	0.654 (0.541-0.767)	0.333	74.6	54.3
2	0.743 (0.643-0.843)	0.300	84.5	55.6
3	0.639 (0.517-0.758)	0.390	78.9	51.4

Influence of PPM values on 3D CT fracture patterns

The average PPM value among three observers was used to represent each patient for assessing fracture stability. The PPM<0.3 group included 27 patients (16 stable and 11 unstable in CT-based classification), while the PPM≥0.3 group comprised 79 patients (19 stable and 60 unstable), demonstrating a significant difference in the distribution of stable or unstable patients (p<0.0008; see Table 5).

**Table 5:** Comparison of two groups based on PPM values in relation to stable and unstable fracture in 3D CT interpretation ^‡^ The chi-square test for PPM evaluation analyzed in the two groups PPM - posterior protrusion measures

PPM		<0.3 n=27	≥0.3 n=79	p-value
Fracture type	Stable	16	19	0.0008‡
	Unstable	11	60	

## Discussion

We propose the integration of a supplementary metric, denoted as posterior protrusion measures (PPM), as an instrumental predictor within the dichotomized 3D CT fracture classification system. This study delineates a significant variance in PPM values between stable and unstable fracture groups. Furthermore, within the realm of subclassification, PPM values exhibit a noteworthy escalation, corresponding to an incremental fragmentation order ranging from two to four parts fractures. In essence, the PPM values potentially offer insights into the corresponding 3D structural configurations. Moreover, two groups predicated on a predefined threshold of 0.3 for PPM have a linear relation with the dichotomized classification of stable and unstable groups (p<0.005).

Challenges with CT dependency

Despite the consensus among most surgeons regarding the accuracy of CT scans in elucidating fracture structures, three noteworthy challenges warrant acknowledgment. Firstly, the accessibility of this modality remains notably constrained for such fractures, particularly outside East Asian countries like Japan, South Korea, and China, as indicated by CT-related publications [18,19]. Secondly, the well-acknowledged drawback of radiation exposure poses a significant concern with this modality [13]. Lastly, there is a prevailing unease that some young surgeons might be gradually losing the proficiency to interpret plain radiographs, especially the lateral view, as an overreliance on CT scans becomes more prevalent.

The rationale for PPM method

Our advocacy for retaining the value of the lateral view of plain radiographs in accessing the 3D CT-based classification system stems from the critical significance of the posterior part of the intertrochanteric region or greater trochanter in fracture classification. Unlike the anteroposterior (AP) view, which might hinder the detection of posterior structural nuances, the lateral view provides a rational basis for utilizing the PPM method. This aligns with other plain radiographic classifications, such as the revised AO/OTA classification system, where the lateral wall thickness of the femur assumes critical importance [10]. While some authors have emphasized the importance of lateral views for postoperative considerations, our study uniquely underscores its relevance in fracture classification [20]. However, it is imperative for surgeons to recognize the limitations of PPM, since it does not serve as a panacea for detecting all unstable cases, indicated by its relatively poor AUC. While PPM quantifies displacement of the posterior fragment, it diverges from the traditional fracture classification system that emphasizes the number of fragments. For instance, substantially displaced two-part fractures might be judged as unstable in PPM, contrary to graphical classifications.

Strengths

The PPM method boasts several strengths in the context of 3D CT classification. Firstly, the PPM study's applicability extends beyond a simple stable-unstable binary classification to encompass the complete seven-class classification. Secondly, it offers simplicity and ease in aiding a surgeon's decision-making process. Thirdly, the method can be employed with various lateral views, including the Lauwenstein view and cross-leg axial view, as demonstrated by our preliminary study on a saw-bone model, which affirmed the applicability of the PPM technique in different radiographic lateral views [21]. Fourthly, it allows for numerical expression, aligning with the severity of fractures, as demonstrated in the sub-classificational study. This contrasts with conventional pertrochanteric fracture classifications like Evan, Jensen, and AO/OTA, which lack the concept of degree of displacement correlating to soft tissue damage.

Limitations

This study is not without its limitations. Firstly, the number of observers remained at three with a single examination, fewer compared to other studies [22,23]. However, PPM, being fundamentally a geometric method, is less likely to depend on subjective judgment, as evidenced by our preliminary article demonstrating high reproducibility among observers [14]. Secondly, the displacement of the greater trochanter may perplex inexperienced residents, with an overlap of the ischial tuberosity in the lateral view. Adequate training by senior surgeons can mitigate this challenge, requiring only a brief period of instruction. Thirdly, there was a discrepancy in the number of patients in subclassification in the current study. Some two-part fractures might have been omitted when treated conservatively, and the number of three-part L and four-part fractures definitely reduced since some have been categorized as "subtrochanteric fracture", not "pertrochanteric fracture" in the surgical database. Fourthly, it is noteworthy that our patient cohort comprises individuals of Asian descent. Therefore, in order to garner comprehensive acknowledgment, it is imperative that this research range be extended to encompass diverse racial backgrounds.

Future direction

In the clinical realm, we advocate for a meticulous analysis of both the AP and lateral radiograph views. Equal emphasis should be placed on the lateral view, appreciating the posterior osseous structures critical for fracture classification. Surgeons must cultivate the ability to image 3D views from two-dimensional plain radiographs, augmented by insights provided by the PPM method. Additionally, there is a need to explore advanced fracture classifications that incorporate the concept of quantifying femoral fracture displacement.

## Conclusions

The present study has illuminated a noteworthy dichotomy in the posterior protrusion measures (PPM) values, effectively discriminating between stable and unstable groups within the ambit of 3D CT fracture classification. Additionally, a discernible upward trajectory in PPM values is observed with the progression of unstableness in the 3D CT classification hierarchy. A pivotal revelation from our findings is the identification of a threshold PPM value of 0.3, delineating a crucial juncture in fracture stability determination.

This groundbreaking methodology, marked by its simplicity in various lateral views among different femoral rotation, stands as a valuable asset in the daily clinical endeavors of orthopedic surgeons. Its profound impact extends to the matter of implant selection and guides the nuanced decision-making process concerning the reduction of displaced fragments, potentially obviating the need for extensive reliance on 3D CT scanning.
